# Functional and Regional Specificity of Noradrenergic Signaling for Encoding and Retrieval of Associative Recognition Memory in the Rat

**DOI:** 10.1523/JNEUROSCI.2408-24.2025

**Published:** 2025-05-01

**Authors:** Stephanie Tran, Gareth R. I. Barker, Mathias L. Mathiasen, John P. Aggleton, Elizabeth Clea Warburton

**Affiliations:** ^1^School of Physiology, Pharmacology and Neuroscience, University of Bristol, Bristol BS8 1TD, United Kingdom; ^2^School of Psychology, Cardiff University, Cardiff CF10 3AT, United Kingdom

**Keywords:** associative recognition, encoding, hippocampus, locus ceruleus, noradrenaline, nucleus reuniens

## Abstract

Recognition of a familiar object in a novel location requires retrieval of the former object–place association and encoding of novel information. Such object-in-place (OiP) memory recruits a neural network including the hippocampus (HPC), medial prefrontal cortex (mPFC), and nucleus reuniens of the thalamus (NRe); however, the underlying cellular mechanisms are not understood. Locus ceruleus (LC) noradrenergic neurons signal novelty; thus here we focused on the contribution of LC–forebrain projections and noradrenaline (NA) receptor subtypes to OiP encoding compared with retrieval, using an arena-based OiP task in male rats. The NRe was found to receive a catecholaminergic input from LC, with the strongest innervation directed to rostral NRe. Interestingly optogenetic inactivation of the LC→NRe pathway impaired OiP retrieval but was without effect on encoding, while inactivation of the LC→HPC selectively impaired encoding. Consistent with this double dissociation, pharmacological blockade of NRe α1-adrenoreceptors selectively impaired memory retrieval, while blockade of HPC β-adrenoreceptors impaired encoding. Finally, pharmacological attenuation of noradrenergic signaling in the NRe and HPC through the infusion of the α2-adrenergic receptor agonist UK 14,304 impaired retrieval and encoding, respectively. Surprisingly, antagonism or agonism of adrenoreceptor subtypes in the mPFC had no effect on memory performance. Together these results reveal the importance of NA within the HPC and NRe for OiP, whereby selectivity of function is achieved via spatially distinct LC output projections and NA receptor subtypes consistent with a modular view of NA function. These results are also important in demonstrating the distinct neuronal mechanisms by which encoding and retrieval are achieved.

## Significance Statement

Noradrenaline projections from the locus ceruleus (LC) have been recognized as providing a novelty signal to the forebrain yet whether this signal is important in mediating different stages of memory is poorly understood. Our results demonstrate that associative recognition memory retrieval is selectively mediated by a direct projection from the LC to the nucleus reuniens of the thalamus (NRe) and by activation of NRe α1- and α2-adrenoreceptors. Conversely encoding is selectively mediated by LC input to the hippocampus (HPC) and by HPC β- and α2-adrenoreceptors. These findings reveal functional and regional specificity of noradrenergic modulation of memory processing in the context of memory circuitry and thus enable the definition of clearer targets for disease-modifying therapies for patients with memory deficits.

## Introduction

Remembering a stimulus such as an object and the location in which it was last encountered is a crucial memory process. Such memories can be formed rapidly in a “one-shot” encoding of an object-in-place (OiP) association, while retrieval of this association enables the rapid detection of a change in our environment. We have previously identified a hippocampal (HPC)–medial prefrontal cortex (mPFC)–nucleus reuniens of the thalamus (NRe) network in which specific neural pathways differentially mediate encoding and retrieval of OiP (Barker et al., 2021), yet the underlying cellular mechanisms by which these processes are mediated are poorly understood.

Stimulus novelty is a key factor in driving memory encoding (Dunsmoor et al., 2022), and exploration of novel objects or environments produces significant increases in neuronal firing within the locus ceruleus (LC) (Sara et al., 1994; Vankov et al., 1995), the origin of forebrain noradrenaline (NA) afferents, suggesting that the LC provides a key signal which drives memory formation. Consistent with this hypothesis, behavioral studies show that activity in the LC→HPC and LC→mPFC projections is critical for spatial memory encoding and contextual fear learning, while blockade of HPC α1-adrenergic or β-adrenergic receptors impairs spatial memory learning (Lemon et al., 2009; Torkaman-Boutorabi et al., 2014; Fan et al., 2022; Tsetsenis et al., 2022). Although the role of NA in encoding is clear, for retrieval it is less so. For example, in one study, retrieval of a contextual fear memory was found to require a decrease in NA release in the HPC (Wilson et al., 2024); in contrast, increased NA in the basolateral amygdala enhanced retrieval (Fukabori et al., 2020). Thus, evidence indicates that NA neurotransmission is involved in both memory encoding and retrieval, yet its role is clearly complex and may differ depending on the brain region and/or task under investigation.

As stated, OiP memory depends on a HPC–NRe–mPFC network (Barker et al., 2021). While the LC–NA projections to HPC and mPFC are numerous and well described (Loughlin et al., 1986; Smith and Greene, 2012), the NRe has been reported to receive only limited input (Lindvall et al., 1974; Swanson and Hartman, 1975; McKenna and Vertes, 2004) despite appearing to have dense adrenoreceptor expression (Palacios and Kuhar, 1982; Sargent-Jones et al., 1985; Boyajian et al., 1987). Thus, it is likely that the density of NA innervation to the NRe is more extensive than previously described and, if so, may have an important role in the neuromodulation of encoding or retrieval of associative recognition memory.

To examine the relationship between forebrain NA and the encoding and retrieval of associative recognition memory, we took a circuit analysis approach using (1) anatomical tracing techniques to map the extent of catecholaminergic projections from the LC to NRe, (2) combined optogenetic and recognition memory testing to assess the importance of LC inputs to the HPC and NRe on encoding and retrieval, and (3) selective pharmacological manipulations of adrenergic receptor subtypes to establish their relative contribution to OiP encoding and retrieval.

## Materials and Methods

### Animals

For the anatomical studies, eight male Lister Hooded rats (Envigo) weighing 297–307 g at the start of experimentation were used. Rats were group housed (2–4 per cage) kept on a 12 h light/dark cycle (light phase, 06:00–18:00). For the behavioral studies, 48 male Lister Hooded rats (Harlan Laboratories) weighing 300–400 g at the start of experimentation were used. For the optogenetic experiments, animals were split into two groups: those that received the control virus, AAV5-CaMKII-EYFP (YFP; *n* = 12), and those that received the virus that expresses the inhibitory opsin, AAV5-CaMKII-eArch3.0-EYFP (Arch; *n* = 12). For the pharmacology experiments, animals were split into two groups: bilateral cannula implanted into the NRe (*n* = 12) and bilateral cannula implanted into the HPC and mPFC (a total of four cannulae per animal; *n* = 12). Rats were group housed (2–4 per cage) and kept on a 12 h light/dark cycle (light phase, 18:00–06:00). All animals had *ad libitum* access to water and standard chow. All animal procedures were conducted in compliance with the Animals (Scientific Procedures) Act (1986).

### Surgical procedures

#### General surgical procedures

Animals were anesthetized using isoflurane (induction 4%, maintenance 2%). The scalp of the animals was shaved before they were positioned in a stereotaxic frame; the incisor bar was adjusted to achieve a flat skull (David Kopf Instruments). Before the start of surgery, animals received eye drops (0.1% sodium hyaluronate; Hycosan) and topical application of both lidocaine (5% m/m; TEVA) and chlorhexidine on the scalp. Following surgery, the skin was sutured, and antibiotic wound powder (2% w/w; Battle) was applied. Immediately postsurgery animals received eye drops (0.1% sodium hyaluronate; Hycosan), subcutaneous injection of 5 ml glucose saline (sodium chloride 0.9% w/v with glucose 5% w/v), intramuscular injection of 0.05 ml Vetergesic (0.3 mg/ml buprenorphine; Ceva Animal Health), and intramuscular injection of 0.1 ml Clamoxyl (150 mg/ml; Zoetis).

#### Injection of anatomical tracers

Each animal received a unilateral injection of an anatomical tracer into the NRe. All tracer injections were given at a 6° angle from the mediolateral (ML) plane. The stereotaxic coordinates were derived from the rat brain atlas of Paxinos and Watson (2007). As the NRe lies directly ventral to the sagittal sinus, ML coordinates used were aimed to target as close to the side of the sagittal sinus as possible. Table 1 shows a list of cases including details of the coordinates used, anatomical tracer used, and the main site of tracer deposit. For pressure injections, fast blue (FB) or cholera toxin B subunit (CTB) were mechanically injected via a 1 µl Hamilton syringe (Hamilton); 55 nl was injected per site at a rate of 20 nl/min. The syringe was left in situ for 3 min prior to injection and 10 min after injection to minimize leakage of tracer. For iontophoretic injections, CTB or FluoroGold (FG) was injected using a glass micropipette (tip diameter, 15–20 µm). A positive pulsed current (2 µA for 6 min followed by 6 µA for 6 min and finally 7 µA for 6 min) was applied using Digital Midgard Precision Current Source iontophoretic pump (Stoelting) on a cycle of 6 s on/6 s off. After the injection period, the glass micropipette was left in situ for 3 min to minimize leakage of tracer. During withdrawal of the micropipette, a negative current was applied. All animals were allowed to recover for 7 d before being killed for subsequent histological processing.

**Table 1.** Overview of individual cases with details of retrograde tracers used and method of injection

Case number	Tracer	Coordinates	Method of injection	Main site of tracer deposit
1	FB	AP, −1.9; ML, sinus; DV, −7.4	Pressure	Rostral NRe
2	CTB	AP, −1.9; ML, sinus; DV, −7.5	Pressure	Rostral NRe
3	CTB	AP, −1.9; ML, sinus; DV, −7.5	Pressure	Rostral NRe
4	CTB	AP, −1.9; ML, sinus; DV, −6.8 (dura)	Iontophoretic	Rostral NRe
5	CTB	AP, −2.4; ML, sinus; DV, −6.9 (dura)	Iontophoretic	Intermediate to caudal NRe
6	FG	AP, −2.6; ML, sinus; DV, −6.8 (dura)	Iontophoretic	Intermediate to caudal NRe
7	CTB	AP, −2.6; ML, sinus; DV, −6.7 (dura)	Iontophoretic	Intermediate to caudal NRe

#### Viral injections and implantation of optical fibers

Animals received a bilateral injection of AAV5-CaMKII-eArchT3.0-EYFP (Arch group) or AAV5-CaMKII-EYFP (YFP group) into the LC. To target the LC, the following coordinates were used: anterior–posterior (AP) −9.6 mm, ML ±1.4 mm, and dorsoventral (DV) −7.4 mm. Each animal received two injections (one in each hemisphere) of virus through a 5 µl Hamilton syringe. Each virus was injected at a rate of 0.2 µl/min using a Micro4 controller infusion pump (World Precision Instruments), attached to the arm of the stereotaxic frame. The needle was left in situ for a further 10 min before being withdrawn. Following injection of the virus, animals were immediately implanted with bilateral optical fibers to target both the NRe and HPC. Therefore, for a given animal, four optical fibers were implanted (two aimed at the NRe and two aimed at the HPC). To implant optical fibers, burr holes were drilled into the skull to allow implantation of optical fiber [core, 200 µm; numerical aperture, 0.22 (MFC 200/240-0.22 SM3 C45 Mono Fiber-optic Cannula); Doric Lenses]. Four stainless steel screws (Plastics One) and dental cement were used to anchor the optical fibers. To target the NRe, animals were implanted with bilateral optical fiber (length, 7 mm) using the following coordinates: AP −1.8 mm, ML ±2 mm, and DV −6.6 mm. All optical fibers were implanted 15° from the ML plane. To target the HPC, animals were implanted with bilateral optical fiber (length, 5.5 mm) using the following coordinates: AP −5.4 mm, ML ±2.7 mm, and DV −2.8 mm. All optical fibers were implanted 25° from the AP plane.

#### Cannula implantation

Burr holes were drilled into the skull to allow implantation of stainless steel guide cannula (26 gauge; Plastics One). Four stainless steel screws (Plastics One) and dental cement were used to anchor the cannula. To target the NRe, animals were implanted with bilateral cannula using the following coordinates: AP −1.8 mm and −2.4 mm, ML ±1.7 mm, and DV −6.4 mm. All cannulae were implanted 15° from the ML plane. To target the HPC or mPFC, animals were implanted with bilateral cannula to target both brain regions. Therefore, for a given animal, four infusion cannulae were implanted (two aimed at the HPC and two aimed at the mPFC). To target the HPC, the coordinates were as follows: AP −4.3 mm, ML ±2.5 mm, and DV −2.8 mm (dura). To target the mPFC, the coordinates were as follows: AP +3.2 mm, ML ±0.75 mm, and DV −3.5 mm. To prevent contamination/cannula blockages, dummy cannulae were inserted into the guide cannula. Animals were singly housed for 7 d postsurgery and given 2 weeks to recover before behavioral testing commenced.

### Behavioral procedures

#### Behavioral apparatus and habituation

Behavioral testing was conducted in a wooden open-topped (90 × 100 × 50 cm) arena with a sawdust-covered floor. One wall of the arena was black, and three other walls were gray in color on one side and surrounded by a black cloth on the north and south side hung from a height of 1.5 m. The room was lit with two floor lamps situated at either side of the arena. A webcam was located above the arena to record behavior. Objects were constructed from DUPLO blocks (Lego) and varied in size (ranging from 16 × 16 × 8 cm to 20 × 20 × 25 cm), color, and shape. Objects were placed 10 cm from the edges of the arena and cleaned with 100% ethanol during the delay period between sample and test and between animals to remove olfactory cues. All animals were handled extensively prior to habituation and then habituated to the behavioral testing setup for 4 d before memory testing.

### Drugs and infusion procedure for cannulation experiments

The following drugs were used: the α2 adrenergic agonist UK 14,304 (2466, Tocris Bioscience); the α2 antagonist RS 79948 (0987, Tocris Bioscience); the α1 antagonist prazosin (0623, Tocris Bioscience); and the β adrenergic antagonist propranolol (0834, Tocris Bioscience). UK 14,304, propranolol, and RS 79949 were dissolved in 0.9% sterile saline solution and infused at the following concentrations: UK 14,304 (10 µM); propranolol (10 µM); and RS 79948 (1 µM). Prazosin was initially dissolved in 100% dimethyl sulfoxide (DMSO); the stock solution was subsequently diluted with 0.9% sterile saline solution, yielding an infusion concentration of 1 µM prazosin in 0.1% DMSO. For the NRe experiments, vehicle control animals received either 0.9% sterile saline solution (UK 14,304 and RS 79948 experiment) or 0.9% sterile saline solution with 0.1% DMSO (prazosin and propranolol experiment). For the HPC–mPFC experiments, vehicle control animals received 0.9% sterile saline solution. Drug doses used were based on published IC50 values (Atlas et al., 1974; Bylund and Snyder, 1976; Lefkowitz et al., 1976; U’Prichard et al., 1978; Greengrass and Bremmer, 1979; van Meel et al., 1981). Drugs were infused via 33 gauge cannula (Plastic Ones) attached to a 25 µl Hamilton syringe by polyethylene tubing. The rate of infusion was controlled using an infusion pump (Harvard Apparatus). For the NRe, animals were infused with 0.3 µl of drug or saline per hemisphere at a rate of 0.3 µl/min. For the HPC, animals were infused with 0.5 µl of the drug or saline per hemisphere at a rate of 0.25 µl/min. For mPFC infusions, animals were infused with 1 µl of the drug or saline per hemisphere at a rate of 0.5 µl/min. Following infusion, cannulae were left in place for 5 min. Infusions were given 15 min before the sample phase to test the effects on encoding or 15 min before the test phase to assess the effects on retrieval.

### Stimulation protocol for optogenetic experiments

Laser light for optical stimulation was generated using a diode laser [Omicron LuxX 515-100 laser (515 nm), Photon Lines]. The laser was attached to a fiber-optic rotary joint with beam splitter (FRJ 1X2i FC-2FC, Doric Lenses) via a fiber-optic patch cord (core, 200 µm; numerical aperture, 0.22; FG200LEA; Thorlabs). Two fiber-optic patch cords (core, 200 µm; numerical aperture, 0.22; FC-CM3; Doric Lenses) were attached to the rotary joint at one end, while the other end was used to connect to the optical implant on the animal's head. The power output of the laser was adjusted so that 10 mW was measured at the tip of each optical fiber. Optical stimulation was either given throughout the length of the sample phase to test the effects on encoding or throughout the length of the test phase to test the effects on retrieval. Laser stimulation was delivered at a frequency of 30 Hz and a duration of 10 ms pulses (50% duty cycle) using a custom protocol on WinLTP (2.20 M/X-Series, WinLTP). Stimulation parameters were chosen based on a previous in vitro electrophysiological study conducted in acute brain slices demonstrating that laser stimulation using the abovementioned parameters resulted in a robust decrease in resting membrane potential (Banks et al., 2021).

### Spontaneous object recognition tasks

The OiP task comprised a sample and test phase, separated by a 3 h delay (Fig. 2*E*). In the sample phase (5 min), each animal was placed in the arena which contained four different objects. Each animal was then allowed to explore the objects before being removed from the arena and placed back into the home cage for the delay. For the test phase (3 min), animals were placed back in the arena which contained the same four objects, but two objects had exchanged positions. Successful OiP memory is demonstrated by the animal preferentially exploring the two moved objects (the novel configuration) compared with the two objects in the same position (familiar configuration).

The novel object recognition (NOR) task comprised a sample and test phase with a 3 h delay (Fig. 2*I*). In the sample phase (5 min), the animal explored four different objects before being removed from the arena and placed in the home cage for the delay. In the test phase, two objects from the sample phase were replaced with novel objects. Intact NOR is demonstrated by greater exploration of the novel over the familiar objects.

The object location (OL) memory task comprised a sample and test phase with a 3 h delay (Fig. 2*J*). For the sample phase (4 min), each animal was placed in the arena which contained two identical objects which they were allowed to explore before being removed from the arena for the delay. Following the delay, the animals were placed back in the arena where the location of one object was changed. Successful OL memory is demonstrated by greater exploration of the familiar object in the new location over the familiar object in the familiar location.

The OiP task with two test phases employed similar methods to the OiP task as described above but consisted of two separate test phases (Fig. 2*G*). At Test Phase 1, two objects, those on either the left or right side of the arena, exchanged positions, and the animals were given 5 min to explore. At Test Phase 2, two objects either on the left or on the right side, exchanged positions, and the animals were given 3 min to explore. If during Test Phase 1, objects to the left exchanged positions, then during Test Phase 2, objects to the right exchanged positions and vice versa. If an animal demonstrates successful OiP memory, it should preferentially explore the two objects which have exchanged positions (the novel configuration) over the two objects which have remained in the same position (familiar configuration). Thus, in Figure 2*G*, at Test Phase 1, animals with intact memory will preferentially explore the objects on the right-hand side of the arena (i.e., the moved objects relative to their position in the sample phase), and at Test Phase 2, animals will preferentially explore the objects on the left-hand side of the arena (i.e., objects which have been moved relative to their positions in Test Phase 1).

### Behavioral scoring

Total object exploration in the sample and test phases was measured using a custom software with the experimenter blind to the experimental condition of the animal. In all tasks, the positioning and/or identity of the objects in the sample and test phases in each task was counterbalanced between the animals. Exploration of an object was measured in seconds and defined as the animal's nose directed toward the object and <2 cm from the object while actively sniffing. Sitting on top of the object or using the object for supported rearing was not scored as exploratory behavior. To measure an animal's ability to discriminate between the novel configuration/object compared with the familiar configuration/object, a discrimination ratio was calculated as follows:

$$\begin{array}{rl} & \mathrm{Discrimination}\;\mathrm{ratio}\\ & \;=\frac{\left(\mathrm{exploration}\;\mathrm{of}\;\mathrm{novel}\;(s)\;\text{–}\;\mathrm{exploration}\;\mathrm{of}\;\mathrm{familiar}\;(s)\right)}{\mathrm{total}\;\mathrm{exploration}\;\mathrm{time}\;(s)}. \end{array}$$

A value of zero indicates no preference for the novel or familiar configuration/object. A positive discrimination ratio value indicates a preference for the novel configuration/object, while a negative value indicates preference for the familiar object/configuration.

### Histology

#### Tissue fixation

On completion of experiments animals received an intraperitoneal injection of sodium pentobarbital (Euthatal, Merial). Animals were transcardially perfused with 0.1 M phosphate-buffered saline (PBS) followed by 4% paraformaldehyde (PFA) in 0.1 M PBS (anatomical tracing or viral injections/optical fiber implantation animals) or 4% formal saline (cannulated animals). Brains were removed and postfixed with PFA for a minimum of 4 h or with formal saline for a minimum of 1 week before being transferred to 25% sucrose in 0.1 M PBS for 24 h.

#### Tissue preparation

Following the tissue fixation procedures outlined above, brains were sectioned using a cryostat (Leica CM3050S) into 40 µm coronal sections. For anatomical tracing and viral injection/optical fiber implantation animals, four series were taken. The first tissue series was directly mounted onto gelatin-subbed slides for cresyl violet staining. The second tissue series was subject to immunohistochemical processing. For cannulated animals, sections were directly mounted onto gelatin-subbed slides and air-dried before staining with cresyl violet. A Leica DM6 B microscope mounted with a Hamamatsu C13440 digital camera was used to image the samples.

#### Immunohistochemical procedure

Immunohistochemical staining was performed on free-floating sections. Sections were washed with 0.1 M PBS (3 × 10 min). Sections were incubated in blocking solution [5% animal serum, 2.5% bovine serum albumin, 0.2% Triton X-100 in 0.1 M PBS (PBST)] for 1 h before incubation with primary antibodies diluted in blocking solution overnight at room temperature. Sections were then washed in 0.1 M PBST (4 × 10 min) before incubation in secondary antibodies diluted in blocking buffer for 2 h at room temperature. Sections were given a final wash with PBS (4 × 10 min) and mounted on gelatin-subbed slides and coverslipped with Fluoromount (Sigma-Aldrich, F4680). The following primary antibodies were used in this study: rabbit anti-TH (tyrosine hydroxylase; 1:1,000, AB152, Chemicon), chicken anti-TH (1:1,000, AB76442, Abcam), chicken anti-GFP (1:1,000, GFP-1020, Aves Labs), and rabbit anti-CTB (1:3,000, C30620, Sigma-Aldrich).

#### Anatomical nomenclature

Anatomical boundaries and nomenclature follow the rat brain atlas of Paxinos and Watson (2007), except for terminology regarding NA-positive neurons which follows the well described nomenclature (Fuxe, 1964; Hokfelt, 1984). To determine the origin of noradrenergic input to the NRe, only noradrenergic cell groups which have previously described projections to the NRe were examined for double-labeled neurons (i.e., those that demonstrate costaining of both the retrograde tracer and TH antibody; McKenna and Vertes, 2004).

#### Cell counts and quantification

For cell counts, the region of interest was determined by the presence of TH-positive cells. All TH-positive cells, retrogradely transported cells, and double-labeled cells within the region of interest were counted for each animal. The Olympus cellSens Dimension Desktop software was used to perform manual cell counts. For cell counts, the region of interest was determined by the presence of TH-positive cells. All TH-positive cells, retrograde tracer-positive cells, and double-labeled cells within the region of interest were counted for each animal. Note the counts were not stereological so should give relative not absolute numbers.

### Experimental design and statistical analysis

The cannulation experiments were run with a cross-over design; thus for a given experiment, each animal received both drug and saline infusions. For the HPC–mPFC implanted animals, saline infusion into the HPC or mPFC was counterbalanced between infusion timing, e.g., for a given drug, if an animal received a presample infusion of saline into the HPC, for the pretest infusion, the same animal would receive saline infusion into the mPFC or vice versa. The optogenetic experiments were run with a cross-over design with each animal tested with both optical stimulation on and off conditions.

In all behavioral experiments, statistical analyses were performed to compare discrimination ratios, sample phase exploration times, and test phase exploration times between conditions. In addition, in all experiments to determine whether the discrimination ratio for each condition was significantly different from chance (a discrimination ratio of zero), one-sample *t* tests were conducted. Alpha was set at 0.05 for all analysis. The IBM SPSS Statistics 25 software (IBM) was used to perform all statistical analysis. Graphs were created using R 3.6.1 (R Core Team). Data are presented as mean ± standard error of the mean (SEM).

## Results

### Catecholaminergic innervation of NRe

**Figure 1.**
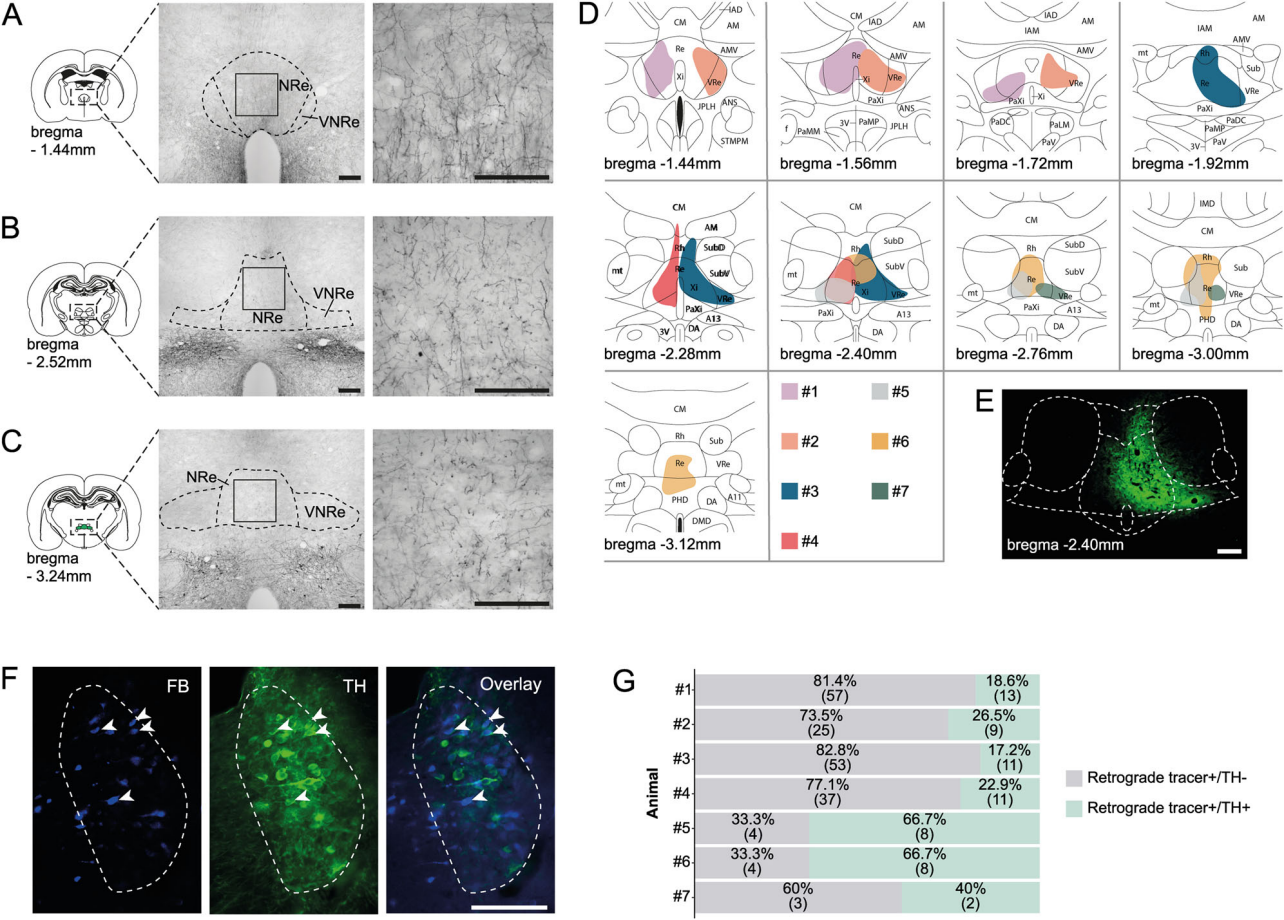
Origin of catecholaminergic input to the NRe. ***A–C***, Distribution of TH-positive fibers in the NRe. Left panel, Schematic of the Paxinos and Watson (2007) brain atlas at three AP levels with the highlighted area (dashed black box) indicating the region in which photomicrographs were taken and distance in millimeter from the bregma. Middle panel, Representative photomicrographs of TH immunoreactive fibers in the thalamus. Right panel, High-magnification photomicrographs of the region indicated by boxes in the middle panel. ***D***, Schematic drawings of retrograde tracer injection spread in each case. Each individual case is color coded, and the Numbers #1–#7 correspond to rostral–caudal injection sites (Table 1). ***E***, Representative Case 3 showing the spread of the CTB tracer in the NRe. ***F***, Fluorescent photomicrographs of Case 1 showing retrogradely transported FB neurons (blue), TH-positive neurons (green), and an overlay of the two images in the LC. Double-labeled neurons highlighted by the white arrowheads. ***G***, Proportion of double-labeled neurons (gray) relative to the number of retrogradely transported cells (green) in A6 for each case. Raw numbers are in brackets, and percentages show the proportion of double-labeled neurons following the injections at different levels (Table 1). Scale bars, 200 µm. A11, A11 dopamine cells; A13, A13 dopamine cells; AHP, anterior hypothalamic area, posterior part; AM, anteromedial thalamic nucleus; AMV, anteromedial thalamic nucleus, ventral part; ANS, accessory neurosecretory nuclei; CM, central medial thalamic nucleus; DA, dorsal hypothalamic area; DMD, dorsomedial hypothalamic nucleus, dorsal part; IAD, interanterodorsal thalamic nucleus; IAM, interanteromedial thalamic nucleus; JLPH, juxtaparaventricular part of lateral hypothalamus; MT, medial terminal nucleus of the accessory optic tract; PaDC, paraventricular hypothalamic nucleus, dorsal cap; PaLM, paraventricular hypothalamic nucleus, lateral magnocellular part; PaMP, paraventricular hypothalamic nucleus, medial parvicellular part; PaXi, paraxiphoid nucleus of thalamus; Pe, periventricular hypothalamic nucleus; PH, posterior hypothalamic nucleus; PHD, posterior hypothalamic area, dorsal part; PT, paratenial thalamic nucleus; PVA, paraventricular thalamic nucleus, posterior part; Re, reuniens thalamic nucleus; Rh, rhomboid thalamic nucleus; Stg, stigmoid hypothalamic nucleus; Sub, submedius thalamic nucleus; SubD, submedius thalamic nucleus, dorsal part; SubV, submedius thalamic nucleus, ventral part; VM, ventromedial thalamic nucleus; VRe, ventral reuniens thalamic nucleus; Xi, xiphoid thalamic nucleus. Figures adapted from Paxinos and Watson (2007).

To visualize the distribution of catecholaminergic innervation to the NRe, an antibody against TH was used. As depicted in Figure 1*A–C*, the entire rostrocaudal axis of the NRe contained TH-immunopositive (TH+) fibers that were fine and spindly in nature. Interestingly the distribution of TH+ fibers in the NRe was nonuniform. At the rostral-most level (Fig. 1*A*), moderate levels of labeled fibers were observed, whereas fewer labeled fibers were observed in the intermediate to caudal levels (Fig. 1*B*,*C*). There was no apparent variation in the density of TH+ fibers in the ML plane.

To examine whether the LC provided a catecholaminergic input to the NRe, we employed retrograde labeling using CTB, FB, or FG combined with TH immunohistochemistry. In all cases analyzed (see Fig. 1*D* for an overview of cases), double-labeled neurons, i.e., neurons immunopositive for both CTB/FB/FG and TH, were observed in the A6-LC (Fig. 1*F*) but were rarely observed from other noradrenergic cell groups analyzed [A7 pontine reticular formation (data not shown)]. In the LC, 63.0% of cells were retrograde+/TH+, and in those cases where the position of the NRe injection was more rostral (Cases 1, 2, 3, 4, 7), there was a greater proportion of double-labeled cells in the LC, compared with the more caudal injections (Cases 5, 6; Fig. 1*D*,*G*). These findings suggest that the LC may provide a stronger catecholaminergic input to the rostral compared with caudal NRe, although further studies are needed to confirm this.

### Dissociation of the role of LC projections to NRe and HPC on OiP encoding and retrieval

**Figure 2.**
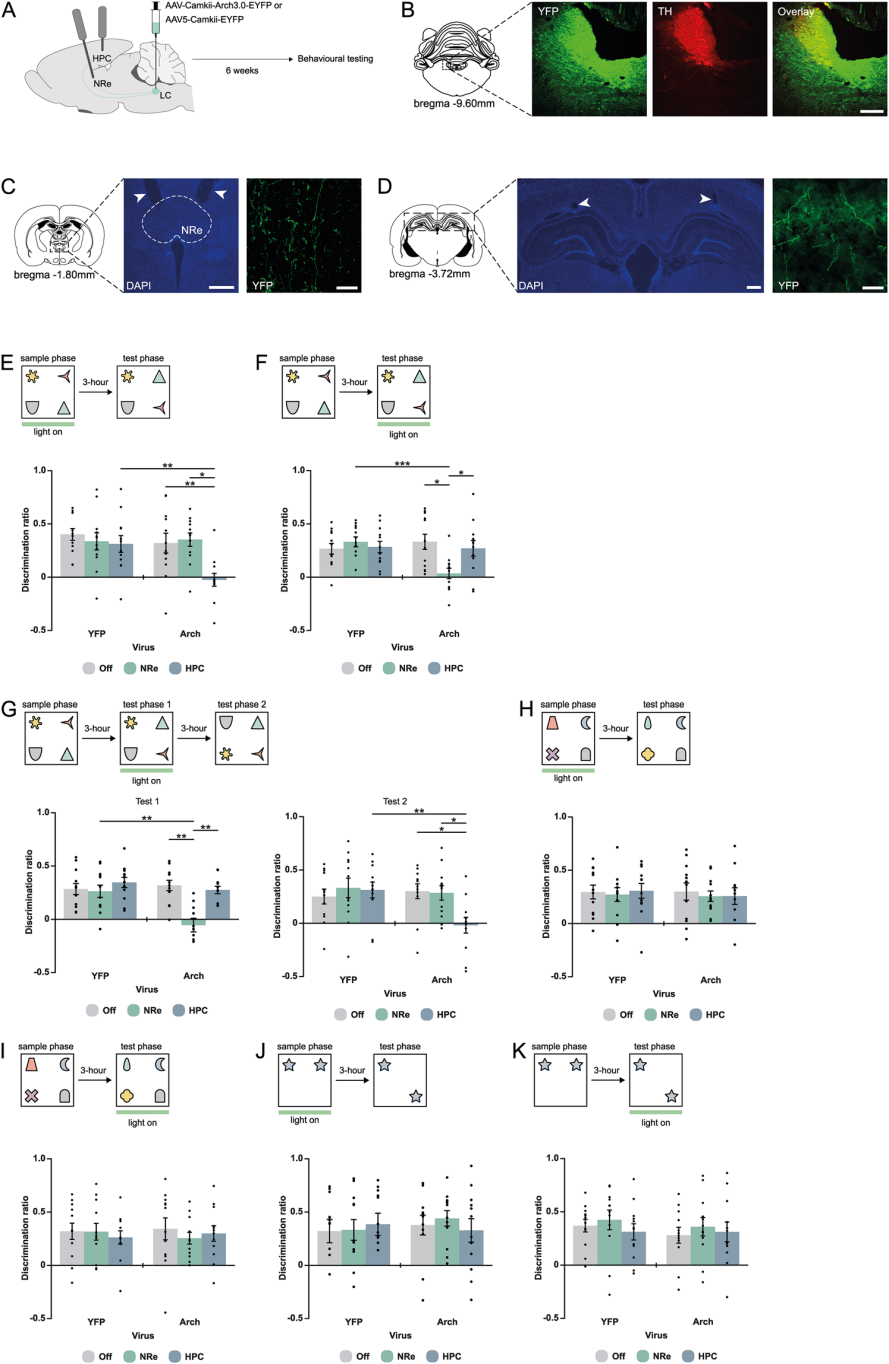
Differential effects of inhibition of LC→NRe and LC→HPC projections on encoding and retrieval of OiP, NOR, and OL memory. ***A***, Schematic of experimental approach for in vivo optogenetic inhibition. ***B***, Representative image of viral expression in the LC. ***C***, Representative image in the NRe showing optrode tracts (left) and Arch3.0-EYFP expression (right). ***D***, A representative image in the HPC showing optrode tracts (left) and Arch3.0-EYFP expression (right). ***E***, OiP performance following light delivery into the NRe and HPC, in the Arch (*n* = 12) and YFP (*n* = 12) animals during the sample phase compared with a no-light “off” condition. ***F***, OiP performance following light delivery into the NRe and HPC, in the Arch (*n* = 12) and YFP (*n* = 12) animals during the test phase compared with a no-light “off” condition. ***G***, OiP performance in the Arch (*n* = 12) and YFP (*n* = 12) animals, in Test Phase 1 and Test Phase 2 with light delivery into the NRe and HPC during Test Phase 1. ***H***, NOR performance following light delivery into the NRe and HPC, in the Arch (*n* = 12) and YFP (*n* = 12) animals during the sample phase compared with a no-light condition (all *F* < 1, n.s.). ***I***, NOR performance following light delivery into the NRe and HPC, in the Arch (*n* = 12) and YFP (*n* = 12) animals during the test phase compared with a no-light condition (all *F* < 1, n.s.). ***J***, OL performance following light delivery into the NRe and HPC, in the Arch (*n* = 12) and YFP (*n* = 12) animals during the sample phase compared with a no-light condition (all *F* < 1, n.s.). ***K***, OL performance following light delivery into the NRe and HPC, in the Arch (*n* = 12) and YFP (*n* = 12) animals during the test phase compared with a no-light condition (all *F* < 1, n.s.). All data represented as mean ± SEM and circles indicate individual animals. *p < 0.05; **p < 0.01 difference between groups. Scale bars, 200 µm.

In view of the observed strong projections from LC to NRe and the previously reported evidence that the LC projection to the HPC is crucial for some forms of memory (Kempadoo et al., 2016; Takeuchi et al., 2016; McNamara and Dupret, 2017; Wagatsuma et al., 2017), we next used specific optogenetic pathway inhibition to assess the functional roles of the LC→NRe and LC→HPC projections on encoding and retrieval of associative recognition memory. Animals received bilateral injection of Arch or YFP into the LC, followed by bilateral implantation of optrodes into the NRe and HPC. Animals were allowed to recover for 6 weeks before behavioral testing commenced (Fig. 2*A*). Following behavioral testing, immunohistochemistry confirmed that viral expression was observed in the LC (Fig. 2*B*) with axonal transport of virus, as well as optrode placement targeting the NRe (Fig. 2*C*) and HPC (Fig. 2*D*).

Figure 2*E* shows the discrimination ratios when light was delivered during the sample phase into the NRe or HPC of the Arch or YFP groups compared with a “light off” condition. We found that light delivery in the Arch-HPC, but not the Arch-NRe group, significantly impaired OiP performance. These results are supported by a significant interaction between stimulation and virus in the ANOVA (*F*_(2,44)_ = 4.06; *p* = 0.024) and Bonferroni-corrected paired *t* test [Arch-off vs Arch-NRe (*p* = 1.00, n.s.); Arch-off vs Arch-HPC (*p* = 0.003); Arch-NRe vs Arch-HPC (*p* = 0.013)]. We next examined the effects of light delivery during the test phase (Fig. 2*F*) and found impairment in the Arch-NRe but not the Arch-HPC group (stimulation by virus interaction; *F*_(2,44)_ = 5.64; *p* = 0.007) Bonferroni-corrected paired *t* test [Arch-off vs NRe (*p* = 0.029); Arch-off vs Arch-HPC (*p* = 1.00); Arch-NRe vs Arch-HPC (*p* = 0.024)].

The double dissociation, i.e., that LC→HPC is required for OiP encoding, while LC→NRe is selectively required for OiP retrieval, suggests a separation of the function of the two pathways. However, encoding of new information and retrieval of old information co-occur during ongoing behavior, which raises the question of whether it is possible to block one process while leaving the other intact. To address this question, we adapted the OiP task, to include two test phases with light stimulation delivered only during Test Phase 1 (Fig. 2*G*, top). If encoding and retrieval are truly mediated by separate neural projections, we hypothesized that LC input to NRe will be required for retrieval of the object–place configurations encoded during the sample phase, as assessed during the first test phase, but not for the encoding the novel object–place configurations encountered during that test phase (i.e., Test Phase 1) which will be dependent on the LC input to HPC. The discrimination ratios following light delivery into the Arch-HPC and Arch-NRe for Test Phase 1 and Test Phase 2 are shown in Figure 2*G*. As expected performance in Test Phase 1 was impaired following light delivery in the Arch-NRe, but not the Arch-HPC condition [ANOVA stimulation × virus; *F*_(2,44)_ = 6.03; *p* = 0.005; Bonferroni-corrected paired *t* test: off vs NRe (*p* = 0.002), off vs HPC (*p* = 1.00), NRe vs HPC (*p* = 0.004)]. In contrast, performance in Test Phase 2 was impaired following light delivery in the Arch-HPC, but not the Arch-NRe condition [ANOVA stimulation × virus; *F*_(2,44)_ = 3.48; *p* = 0.040; Bonferroni-corrected post hoc analysis: off vs Arch-NRe (*p* = 1.00), off vs Arch-HPC (*p* = 0.043), Arch-NRe vs Arch-HPC (*p* = 0.020)].

Finally, we examined the effects of optogenetic inhibition of the LC→HPC and LC→NRe pathways during the sample or test phases of the NOR or OL tasks (Fig. 2*H*–*K*) and no impairments in performance were observed. Further analysis revealed that overall exploration levels in all tasks were not affected (Table 2). In addition, all observations were confirmed by comparing performance against chance, i.e., discrimination of zero (Table 3).

**Table 2.** Mean exploration time ± SEM in the sample and test phases of animals involved in optogenetic experiments

Figure and task	Stimulation timing	Virus	Stimulation condition	Exploration in sample phase (s)	Statistical analysis of sample phase	Exploration in Test Phase 1 (s)	Statistical analysis of Test Phase 1	Exploration in Test Phase 2 (s)	Statistical analysis of Test Phase 2
Figure 2, *E* and *F* OiP	Encoding	YFP	Off	57.9 ± 4.18	Stimulation condition × virus (*F*_(2,44)_ = 2.70; *p* = 0.079) Main effect of stimulation condition (*F*_(2,44)_ = 1.87; *p* = 0.166) Main effect of virus (*F*_(1,22)_ = 0.831; *p* = 0.372)	32.0 ± 4.10	Stimulation condition × virus (*F*_(1.54,33.9)_ = 0.572; *p* = 0.526) Main effect of stimulation condition (*F*_(1.54,33.9)_ = 1.69; *p* = 0.204) Main effect of virus (*F*_(1,22)_ = 0.124; *p* = 0.728)		
			NRe	49.7 ± 4.76		28.2 ± 3.16			
			HPC	41.9 ± 2.52		28.7 ± 3.03			
		Arch	Off	45.5 ± 3.90		31.1 ± 3.29			
			NRe	47.5 ± 3.52		29.9 ± 1.98			
			HPC	47.0 ± 4.29		24.6 ± 3.01			
	Retrieval	YFP	Off	71.5 ± 5.54	Stimulation condition × virus (*F*_(2,44)_ = 0.687; *p* = 0.508) Main effect of stimulation condition (*F*_(2,44)_ = 0.146; *p* = 0.865) Main effect of virus (*F*_(1,22)_ = 0.907; *p* = 0.351)	31.8 ± 4.17	Stimulation condition × virus (*F*_(2,44)_ = 1.11; *p* = 0.338) Main effect of stimulation condition (*F*_(2,44)_ = 0.438; *p* = 0.648) Main effect of virus (*F*_(1,22)_ = 0.004; *p* = 0.950)		
			NRe	68.0 ± 5.34		33.9 ± 4.28			
			HPC	66.3 ± 3.55		28.8 ± 3.28			
		Arch	Off	62.3 ± 4.15		32.6 ± 2.77			
			NRe	65.1 ± 4.39		29.7 ± 3.22			
			HPC	64.1 ± 3.00		31.4 ± 3.48			
Figure 2*G* OiP (two test phases)		YFP	Off	71.0 ± 4.36	Stimulation condition × virus (*F*_(2,44)_ = 0.006; *p* = 0.994) Main effect of stimulation condition (*F*_(2,44)_ = 0.273; *p* = 0.762) Main effect of virus (*F*_(1,22)_ = 0.166; *p* = 0.688)	48.4 ± 4.40	Stimulation condition × virus (*F*_(2,44)_ = 0.184; *p* = 0.833) Main effect of stimulation condition (*F*_(2,44)_ = 0.014; *p* = 0.986) Main effect of virus (*F*_(1,22)_ = 3.66; *p* = 0.069)	34.9 ± 2.22	Stimulation condition × virus (*F*_(2,44)_ = 0.663; *p* = 0.521) Main effect of stimulation condition (*F*_(2,44)_ = 0.006; *p* = 0.994) Main effect of virus (*F*_(1,22)_ = 0.232; *p* = 0.635)
			NRe	72.5 ± 5.37		47.8 ± 4.49		38.6 ± 3.41	
			HPC	69.8 ± 6.41		45.6 ± 3.54		37.4 ± 2.71	
		Arch	Off	68.2 ± 5.71		55.3 ± 5.49		37.6 ± 3.83	
			NRe	70.6 ± 3.42		54.5 ± 5.35		34.3 ± 1.92	
			HPC	67.6 ± 4.74		56.8 ± 4.33		35.9 ± 3.20	
Figure 2, *H* and *I* Object recognition	Encoding	YFP	Off	62.7 ± 3.62	Stimulation condition × virus (*F*_(2,42)_ = 0.041; *p* = 0.959) Main effect of stimulation condition (*F*_(2,42)_ = 1.84; *p* = 0.171) Main effect of virus (*F*_(1,21)_ = 0.005; *p* = 0.947)	36.9 ± 2.81	Stimulation condition × virus (*F*_(2,42)_ = 0.671; *p* = 0.517) Main effect of stimulation condition (*F*_(2,42)_ = 1.77; *p* = 0.183) Main effect of virus (*F*_(1,21)_ = 1.05; *p* = 0.318)		
			NRe	64.8 ± 2.59		41.8 ± 2.90			
			HPC	59.4 ± 3.13		36.0 ± 2.75			
		Arch	Off	61.3 ± 5.26		36.3 ± 3.08			
			NRe	63.2 ± 3.05		41.8 ± 5.23			
			HPC	59.1 ± 4.52		43.1 ± 2.19			
	Retrieval	YFP	Off	67.2 ± 4.99	Stimulation condition × virus (*F*_(2,44)_ = 0.919; *p* = 0.407) Main effect of stimulation condition (*F*_(2,44)_ = 2.95; *p* = 0.063) Main effect of virus (*F*_(1,22)_ = 0.033; *p* = 0.858)	39.6 ± 3.11	Stimulation condition × virus interaction (*F*_(2,44)_ = 0.035; *p* = 0.966) Main effect of stimulation condition (*F*_(2,44)_ = 0.623; *p* = 0.541) Main effect of virus (*F*_(1,22)_ = 0.869; *p* = 0.361)		
			NRe	79.5 ± 3.95		35.0 ± 4.33			
			HPC	72.0 ± 4.82		36.9 ± 4.04			
		Arch	Off	69.5 ± 5.31		43.0 ± 3.33			
			NRe	74.8 ± 4.03		39.5 ± 4.71			
			HPC	77.0 ± 4.08		39.4 ± 4.61			
Figure 2, *J* and *K* OL	Encoding	YFP	Off	39.8 ± 3.74	Stimulation condition × virus (*F*_(2,44)_ = 0.828; *p* = 0.444) Main effect of stimulation condition (*F*_(2,44)_ = 0.066; *p* = 0.936) Main effect of virus (*F*_(1,22)_ = 0.006; *p* = 0.937)	27.3 ± 3.05	Stimulation condition × virus interaction (*F*_(2,44)_ = 0.047; *p* = 0.954) Main effect of stimulation condition (*F*_(2,44)_ = 0.098; *p* = 0.907) Main effect of virus (*F*_(1,22)_ = 4.08; *p* = 0.056)		
			NRe	36.8 ± 4.13		26.5 ± 2.51			
			HPC	39.0 ± 4.07		25.7 ± 2.72			
		Arch	Off	37.2 ± 1.81		31.6 ± 2.76			
			NRe	40.1 ± 3.06		32.1 ± 2.64			
			HPC	39.3 ± 3.19		31.3 ± 2.03			
	Retrieval	YFP	Off	39.8 ± 4.46	Stimulation condition × virus (*F*_(2,44)_ = 0.268; *p* = 0.766) Main effect of stimulation condition (*F*_(2,44)_ = 0.662; *p* = 0.521) Main effect of virus (*F*_(1,22)_ = 0.011; *p* = 0.919)	28.4 ± 2.93	Stimulation condition × virus (*F*_(2,44)_ = 0.753; *p* = 0.872) Main effect of stimulation condition (*F*_(2,44)_ = 0.027; *p* = 0.974) Main effect of virus (*F*_(1,22)_ = 0.083; *p* = 0.775)		
			NRe	41.2 ± 4.17		28.2 ± 3.85			
			HPC	38.9 ± 2.71		30.3 ± 2.40			
		Arch	Off	38.2 ± 2.83		28.2 ± 2.36			
			NRe	42.3 ± 3.19		29.3 ± 2.74			
			HPC	40.8 ± 3.64		25.6 ± 2.69			

**Table 3.** Analysis of performance against chance of animals involved in optogenetic experiment

Figure and task	Stimulation timing	Virus	Stimulation condition	Statistical analysis comparing performance against chance in Test Phase 1	Statistical analysis comparing performance against chance in Test Phase 2
Figure 2, *E* and *F* OiP	Encoding	YFP	Off	*t*_(11)_ = 7.28; *p* < 0.001	
			NRe	*t*_(11)_ = 4.17; *p* = 0.002	
			HPC	*t*_(11)_ = 3.95; *p* = 0.002	
		Arch	Off	*t*_(11)_ = 3.44; *p* = 0.006	
			NRe	*t*_(11)_ = 5.63; *p* < 0.001	
			HPC	*t*_(11)_ = −0.425; *p* = 0.679	
	Retrieval	YFP	Off	*t*_(11)_ = 5.30; *p* < 0.001	
			NRe	*t*_(11)_ = 7.12; *p* < 0.001	
			HPC	*t*_(11)_ = 5.35; *p* < 0.001	
		Arch	Off	*t*_(11)_ = 4.66; *p* = 0.001	
			NRe	*t*_(11)_ = 0.704; *p* = 0.496	
			HPC	*t*_(11)_ = 3.66; *p* = 0.004	
Figure 2*G* OiP (two test phases)		YFP	Off	*t*_(11)_ = 5.52; *p* < 0.001	*t*_(11)_ = 3.59; *p* = 0.004
			NRe	*t*_(11)_ = 4.17; *p* = 0.001	*t*_(11)_ = 3.63; *p* = 0.004
			HPC	*t*_(11)_ = 3.95; *p* = 0.001	*t*_(11)_ = 4.22; *p* = 0.001
		Arch	Off	*t*_(11)_ = 6.54; *p* < 0.001	*t*_(11)_ = 4.35; *p* = 0.001
			NRe	*t*_(11)_ = −0.853; *p* = 0.412	*t*_(11)_ = 4.20; *p* = 0.001
			HPC	*t*_(11)_ = 7.97; *p* < 0.001	*t*_(11)_ = −2.49; *p* = 0.808
Figure 2, *H* and *I* Object recognition	Encoding	YFP	Off	*t*_(11)_ = 4.59; *p* = 0.001	
			NRe	*t*_(11)_ = 4.18; *p* = 0.002	
			HPC	*t*_(11)_ = 4.40; *p* = 0.001	
		Arch	Off	*t*_(11)_ = 3.72; *p* = 0.003	
			NRe	*t*_(11)_ = 5.27; *p* < 0.001	
			HPC	*t*_(10)_ = 3.31; *p* = 0.008	
	Retrieval	YFP	Off	*t*_(11)_ = 4.22; *p* = 0.001	
			NRe	*t*_(11)_ = 4.04; *p* = 0.002	
			HPC	*t*_(11)_ = 4.21; *p* = 0.001	
		Arch	Off	*t*_(11)_ = 3.35; *p* = 0.007	
			NRe	*t*_(11)_ = 4.62; *p* = 0.001	
			HPC	*t*_(11)_ = 4.11; *p* = 0.002	
Figure 2, *J* and *K* OL	Encoding	YFP	Off	*t*_(11)_ = 2.93; *p* = 0.014	
			NRe	*t*_(11)_ = 3.44; *p* = 0.006	
			HPC	*t*_(11)_ = 3.67; *p* = 0.004	
		Arch	Off	*t*_(11)_ = 4.08; *p* = 0.002	
			NRe	*t*_(11)_ = 6.10; *p* < 0.001	
			HPC	*t*_(11)_ = 2.97; *p* = 0.013	
	Retrieval	YFP	Off	*t*_(11)_ = 6.23; *p* < 0.001	
			NRe	*t*_(11)_ = 4.60; *p* = 0.001	
			HPC	*t*_(11)_ = 4.11; *p* = 0.002	
		Arch	Off	*t*_(11)_ = 3.75; *p* = 0.003	
			NRe	*t*_(11)_ = 4.19; *p* = 0.002	
			HPC	*t*_(11)_ = 3.35; *p* = 0.006	

### α1-, α2-, and β-adrenergic receptors play a regionally specific role in OiP memory

Given the differential roles of LC innervation of the HPC and NRe on OiP encoding and retrieval, we next examined the role of specific adrenergic receptor subtypes in the NRe and HPC. In these experiments, we also included a group in which infusions were made into the mPFC for a number of reasons: (1) neuronal activity in the mPFC is key for associative recognition memory encoding and retrieval (Barker et al., 2007; Barker and Warburton, 2011; Benn et al., 2016); (2) the mPFC receives a significant NA input (Chandler and Waterhouse, 2012; Agster et al., 2013; Cerpa et al., 2019); (3) we have previously found the selective role of D1/D5 receptors in the mPFC for OiP memory encoding, but not retrieval (Savalli et al., 2015); and (4) the role of NA receptors in the mPFC has not yet been examined.

**Figure 3.**
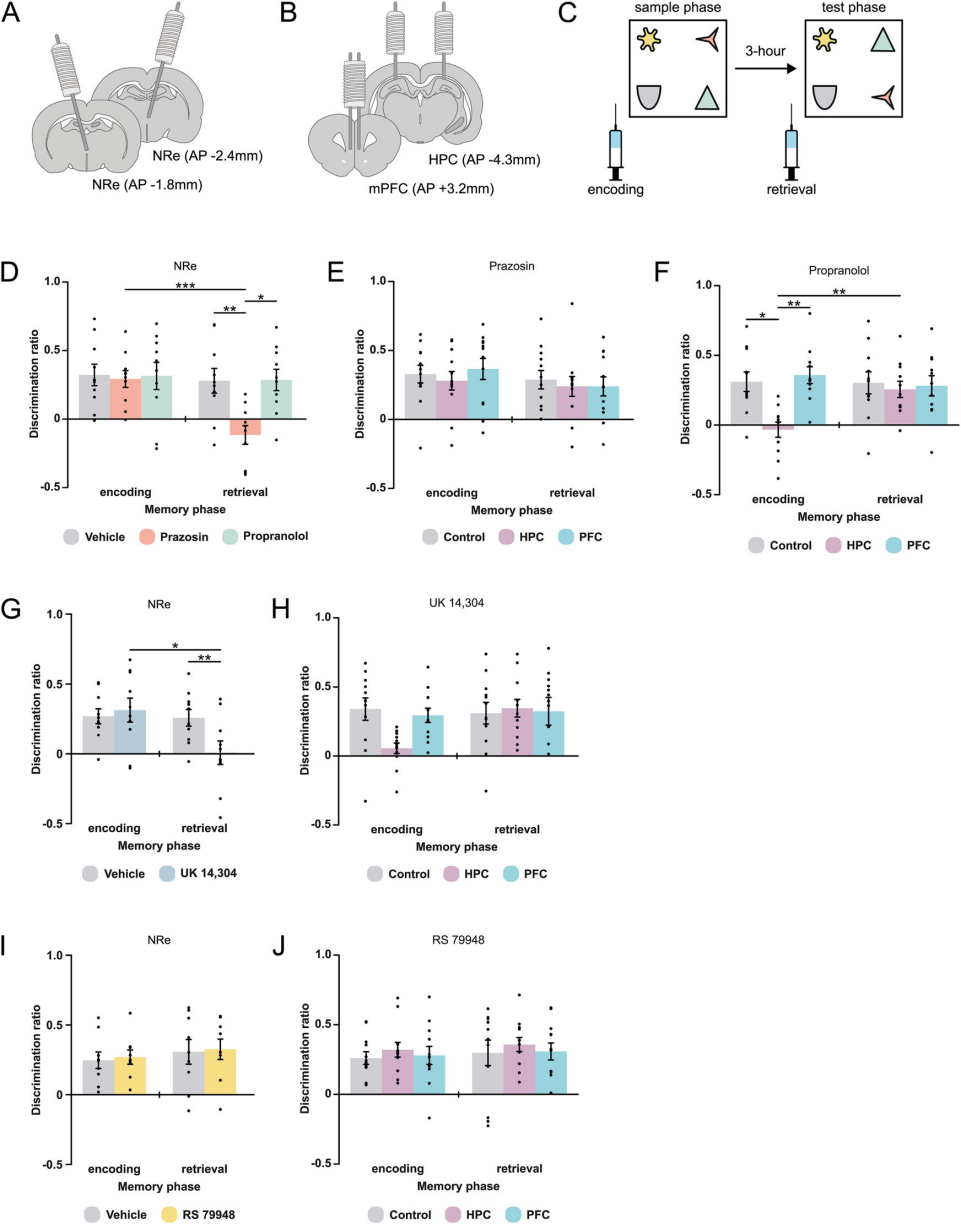
The differential role of adrenergic receptors on OiP encoding and retrieval. ***A***,***B***, Schematic of experimental approach for intracerebral administration of specific adrenergic receptor agonists/antagonists. ***C***, Schematic representation of the OiP task. ***D***, The effects of intra-NRe administration of prazosin or propranolol prior to the sample phase or test phase (*n* = 10). ***E***, The effects of intra-HPC or intra-mPFC infusion of prazosin prior to the sample or test phase (*n* = 12). ***F***, The effect of administration of propranolol into the HPC or mPFC before the sample or test phase (*n* = 11). ***G***, The effect of administration of UK 14,304 into the NRe before the sample or test phase (*n* = 10). ***H***, The effect of administration of UK 14,304 into the HPC or PFC either before the sample or test phase. ***I***, The effect of administration of RS79948 in the NRe before the sample or test. ***J***, The effect of RS 79948 into the HPC or mPFC before the sample or test. Data represented as mean ± SEM and circles indicate individual animals; *p < 0.05; **p < 0.01; ***p < 0.001.

Two groups of animals received surgery to bilaterally implant chronically indwelling cannulae aimed, in one group, at the NRe only (Fig. 3*A*) or, in the second group, at both the HPC and mPFC (Fig. 3*B*). The cannulae allowed local administration of selective receptor antagonists prazosin (α1-adrenergic antagonist), propranolol (β-adrenergic antagonist), UK 14,304 (α2-adrenergic receptor agonist), or RS79948 (α2-adrenergic receptor antagonist), either before the sample phase, to investigate effects on encoding, or before the test phase to investigate effects on retrieval (Fig. 3*C*).

Intra-NRe administration of either prazosin or propranolol before the sample phase had no effect on OiP performance (Fig. 3*D*); however, when the infusions were delivered prior to the test phase, prazosin, but not propranolol, significantly impaired performance, as confirmed by significant drug × infusion timing interaction [*F*_(2,36)_ = 4.09; *p* = 0.025; Bonferroni-corrected post hoc *t* test: pretest vehicle vs prazosin (*p* = 0.006); prazosin vs propranolol (*p* = 0.011); vehicle vs propranolol (*p* = 1.00, n.s.)].

Local infusion of prazosin into either the HPC or mPFC, presample or pretest, had no effect (Fig. 3*E*; region × infusion timing; *F*_(2,44)_ = 0.222, n.s.). In contrast, presample intra-HPC administration of propranolol produced a significant memory disruption while presample intra-mPFC infusions had no effect [Fig. 3*F*; region × infusion timing; *F*_(2,40)_ = 3.73; *p* = 0.033; Bonferroni-corrected post hoc *t* test presample infusion timepoint: vehicle vs HPC (*p* = 0.017); vehicle vs mPFC (*p* = 1.00); HPC vs mPFC (*p* = 0.006)]. Together these results show that α1-adrenergic receptors in the NRe are critical for retrieval, while β-adrenergic receptors in HPC are critical for OiP encoding.

In the final series of experiments, we investigated the effects of inhibiting or stimulating NA release, by local infusion of the α2-adrenergic receptor agonist (UK 14,304) or antagonist (RS 79948). As α2-adrenergic receptors exist, although not exclusively, as autoreceptors, located presynaptically on the terminals of noradrenergic neurons (Langer, 1974; Milner et al., 1998; Starke, 2001). Previous microdialysis studies have shown that UK 14,304 infusions cause a robust decrease in NA levels (van Veldhuizen et al., 1993; Dalley and Stanford, 1995; Ferry et al., 2015), while infusion of RS 79948 results in a robust increase in NA (Fernández-Pastor and Meana, 2002; Horrillo et al., 2019). Here we found that intra-NRe infusion of UK 14,304 before the test, but not before the sample phase, significantly impaired discrimination [Fig. 3*G*; drug × infusion timing; *F*_(1,18)_ = 6.29; *p* = 0.022; Bonferroni-corrected post hoc *t* test presample: vehicle vs UK 14,304 (*t*_(9)_ = −0.462, n.s.); pretest: vehicle vs UK 14,304 (*t*_(9)_ = 3.62; *p* = 0.006)]. In contrast, intra-HPC infusion of UK 14,304 prior to the sample, but not test, impaired discrimination, while infusions into the mPFC had no effect (Fig. 3*H*; region × infusion timing; *F*_(2,44)_ = 2.67; *p* = 0.080, n.s.). Comparisons against chance showed that presample infusion into the HPC significantly impaired discrimination (*t*_(11)_ = 1.45; *p* = 0.176), while all other groups significantly discriminated vehicle [presample (*t*_(11)_ = 4.19; *p* = 0.002); pretest (*t*_(11)_ = 3.98; *p* = 0.002)]; mPFC [presample (*t*_(11)_ = 5.69; *p* < 0.001); pretest (*t*_(11)_ = 3.20; *p* = 0.008)], and HPC [pretest (*t*_(11)_ = 5.41; *p* < 0.001)]. When we tested the effect of RS 79948 into the NRe, HPC, or mPFC, we found no effects on memory performance irrespective of the brain region or timing of infusion (Fig. 3*I*,*J*) confirmed by ANOVA [NRe: drug × infusion timing interaction (*F*_(1,16)_ = 0.001; *p* = 0.978); HPC vs mPFC: region × infusion timing (*F*_(2,44)_ = 0.003; *p* = 0.997)].

Analysis of total object exploration during the sample and test phases indicated overall exploration levels in all tasks were not affected. While some analyses revealed significant main effect of infusion timing, further analysis revealed that this effect was importantly independent of infusion region and due to differences observed in exploration times when either presample or pretest infusions were given (Tables 4, 5). In addition, analysis comparing performance against chance confirmed these observations (Tables 6, 7). Together, these results support our conclusions that successful OiP encoding and retrieval requires release of NA in the HPC and NRe, respectively.

**Table 4.** Mean exploration times ± SEM in the sample and test phases of NRe-infused animals

Figure	Infusion timing	Condition	Exploration in sample phase(s)	Statistical analysis of sample phase	Exploration in test phase(s)	Statistical analysis of test phase
Figure 3*D* Prazosin and propranolol	Presample	Vehicle	74.2 ± 10.7	Drug × infusion timing (*F*_(2,36)_ = 0.085; *p* = 0.918) Main effect of drug (*F*_(2,36)_ = 1.24; *p* = 0.301) Main effect of infusion timing (*F*_(1,18)_ = 6.00; *p* = 0.025)	47.5 ± 5.29	Drug × infusion timing (*F*_(2,36)_ = 0.268; *p* = 0.766) Main effect of drug (*F*_(2,36)_ = 0.295; *p* = 0.747) Main effect of infusion timing (*F*_(1,18)_ = 0.267; *p* = 0.612)
		Prazosin	83.2 ± 6.40		47.5 ± 7.90	
		Propranolol	84.7 ± 6.30		49.5 ± 6.48	
	Pretest	Vehicle	89.7 ± 4.37		41.7 ± 2.54	
		Prazosin	92.9 ± 7.74		48.2 ± 6.12	
		Propranolol	99.6 ± 3.46		45.4 ± 3.91	
Figure 3*G* UK 14,304	Presample	Vehicle	76.9 ± 6.05	Drug × infusion timing (*F*_(1,18)_ = 0.516; *p* = 0.482) Main effect of drug (*F*_(1,18)_ = 0.062; *p* = 0.806) Main effect of infusion timing (*F*_(1,18)_ = 4.02; *p* = 0.060)	38.2 ± 3.23	Drug × infusion timing (*F*_(1,18)_ = 0.026; *p* = 0.874) Main effect of drug (*F*_(1,18)_ = 0.203; *p* = 0.658) Main effect of infusion timing (*F*_(1,18)_ = 4.75; *p* = 0.043)
		UK 14,304	79.3 ± 7.37		37.1 ± 3.29	
	Pretest	Vehicle	93.8 ± 4.68		51.6 ± 5.71	
		UK 14,304	88.8 ± 5.05		46.4 ± 6.42	
Figure 3*I* RS79488	Presample	Vehicle	85.5 ± 5.19	Drug × infusion timing (*F*_(1,16) _= 0.293; *p* = 0.596) Main effect of drug (*F*_(1,16) _= 1.89; *p* = 0.188) Main effect of infusion timing (*F*_(1,16)_ = 10.3; *p* = 0.006)	50.5 ± 4.79	Drug × infusion timing (*F*_(1,16)_ = 0.521; *p* = 0.481) Main effect of drug (*F*_(1,16) _= 0.001; *p* = 0.982) Main effect of infusion timing (*F*_(1,16)_ = 3.33; *p* = 0.087)
		RS79488	76.5 ± 10.4		48.3 ± 7.33	
	Pretest	Vehicle	59.2 ± 4.44		44.6 ± 2.77	
		RS79488	54.7 ± 4.57		42.0 ± 3.27	

**Table 5.** Mean exploration times ± SEM in the sample and test phases of HPC or mPFC-infused animals

Figure and drug	Infusion timing	Drug condition	Exploration in sample phase(s)	Statistical analysis of sample phase	Exploration in test phase(s)	Statistical analysis of test phase
Figure 3*E* Prazosin	Presample	Vehicle	87.0 ± 5.33	Infusion region × infusion timing (*F*_(2,44) _= 1.31; *p* = 0.281) Main effect of infusion region (*F*_(2, 44)_ = 1.09; *p* = 0.345) Main effect of infusion timing (*F*_(1,22) _= 2.10; *p* = 0.161)	53.1 ± 4.12	Infusion region × infusion timing (*F*_(2, 44)_ = 1.38; *p* = 0.262) Main effect of infusion region (*F*_(2,44) _= 0.481; *p* = 0.621) Main effect of infusion timing (*F*_(1,22) _= 1.66; *p* = 0.211)
		HPC	90.9 ± 6.88		51.2 ± 4.13	
		mPFC	76.7 ± 6.23		47.0 ± 3.82	
	Pretest	Vehicle	92.5 ± 6.57		52.6 ± 3.01	
		HPC	93.8 ± 4.86		57.7 ± 3.66	
		mPFC	94.0 ± 3.65		56.5 ± 4.85	
Figure 3*F* Propranolol	Presample	Vehicle	52.4 ± 4.05	Infusion region × infusion timing (*F*_(2,40)_ = 0.868; *p* = 0.482) Main effect of infusion region (*F*_(2,40)_ = 1.63; *p* = 0.208) Main effect of infusion timing (*F*_(1,20)_ = 2.38; *p* = 0.138)	34.9 ± 3.90	Infusion region × infusion timing (*F*_(2,40)_ = 0.915; *p* = 0.409) Main effect of infusion region (*F*_(2,40)_ = 1.81; *p* = 0.176) Main effect of infusion timing (*F*_(1,20)_ = 7.43; *p* = 0.013)
		HPC	54.5 ± 4.15		28.7 ± 2.98	
		mPFC	50.2 ± 4.69		27.0 ± 2.62	
	Pretest	Vehicle	55.9 ± 5.97		38.5 ± 5.36	
		HPC	71.6 ± 8.48		40.6 ± 5.16	
		mPFC	61.6 ± 10.0		35.0 ± 5.47	
Figure 3*H* UK 14,304	Presample	Vehicle	71.9 ± 5.65	Infusion region × infusion timing (*F*_(2,44) _= 0.341; *p* = 0.713) Main effect of infusion region (*F*_(2,44) _= 0.423; *p* = 0.658) Main effect of infusion timing (*F*_(1,22) _= 2.68; *p* = 0.116)	46.9 ± 4.07	Infusion region × infusion timing (*F*_(1.45, 31.9) _= 1.82; *p* = 0.175) Main effect of infusion region (*F*_(1.45, 31.9) _= 0.571; *p* = 0.517) Main effect of infusion timing (*F*_(1,22) _= 0.491; *p* = 0.037)
		HPC	69.4 ± 4.97		46.9 ± 3.69	
		mPFC	65.6 ± 5.87		40.8 ± 3.89	
	Pretest	Vehicle	59.3 ± 4.07		31.3 ± 3.72	
		HPC	60.0 ± 5.61		36.7 ± 3.77	
		mPFC	59.1 ± 3.93		36.9 ± 4.85	
Figure 3*J* RS79488	Presample	Vehicle	69.4 ± 5.17	Infusion region × infusion timing (*F*_(2,44) _= 0.516; *p* = 0.600) Main effect of infusion region (*F*_(2,44) _= 0.026; *p* = 0.974) Main effect of infusion timing (*F*_(1,22) _= 4.83; *p* = 0.039)	36.1 ± 3.77	Infusion region × infusion timing (*F*_(2, 44)_* *= 0.003; *p* = 0.997 Main effect of infusion region (*F*_(2,44) _= 0.465; *p* = 0.631) Main effect of infusion timing (*F*_(1,22) _= 0.499; *p* = 0.487)
		HPC	71.1 ± 4.19		38.5 ± 4.83	
		mPFC	67.3 ± 5.08		39.6 ± 3.61	
	Pretest	Vehicle	60.4 ± 3.72		40.9 ± 3.00	
		HPC	57.2 ± 4.97		42.5 ± 2.69	
		mPFC	62.6 ± 5.51		39.4 ± 4.13	

**Table 6.** Analysis of performance against chance of NRe-infused animals

Figure	Infusion timing	Condition	Statistical analysis of sample phase
Figure 3*D* Prazosin and propranolol	Presample	Vehicle	*t*_(9) _= −4.10; *p* = 0.003
		Prazosin	*t*_(9) _= 4.48; *p* = 0.001
		Propranolol	*t*_(9) _= 3.17; *p* = 0.011
	Pretest	Vehicle	*t*_(9) _= 3.10; *p* = 0.013
		Prazosin	*t*_(9) _= −1.70; *p* = 0.123
		Propranolol	*t*_(9) _= 3.66; *p* = 0.005
Figure 3*G* UK 14,304	Presample	Vehicle	*t*_(9) _= 5.05; *p* = 0.001
		UK 14,304	*t*_(9) _= 3.64; *p* = 0.005
	Pretest	Vehicle	*t*_(9) _= 4.32; *p* = 0.002
		UK 14,304	*t*_(9) _= 0.092; *p* = 0.928
Figure 3*I* RS79488	Presample	Vehicle	*t*_(8) _= 4.17; *p* = 0.003
		RS79488	*t*_(8) _= 5.25; *p* = 0.001
	Pretest	Vehicle	*t*_(8) _= 3.47; *p* = 0.008
		RS79488	*t*_(8) _= 4.44; *p* = 0.002

**Table 7.** Analysis of performance against chance of HPC or mPFC-infused animals

Figure and drug	Infusion timing	Drug condition	Statistical analysis comparing performance against chance
Figure 3*E* Prazosin	Presample	Vehicle	*t*_(11)_ = 5.13; *p* < 0.001
		HPC	*t*_(11)_ = 4.18; *p* = 0.002
		mPFC	*t*_(11)_ = 4.78; *p* = 0.001
	Pretest	Vehicle	*t*_(11)_ = 4.31; *p* = 0.001
		HPC	*t*_(11)_ = 3.32; *p* = 0.007
		mPFC	*t*_(11)_ = 3.47; *p* = 0.005
Figure 3*F* Propranolol	Presample	Vehicle	*t*_(10)_ = 4.45; *p* = 0.001
		HPC	*t*_(10)_ = −6.15; *p* = 0.553
		mPFC	*t*_(10)_ = 5.10; *p* < 0.001
	Pretest	Vehicle	*t*_(10)_ = 3.82; *p* = 0.003
		HPC	*t*_(10)_ = 4.40; *p* = 0.001
		mPFC	*t*_(10)_ = 3.88; *p* = 0.003
Figure 3*H* UK 14,304	Presample	Vehicle	*t*_(11)_ = 4.19; *p* = 0.002
		HPC	*t*_(11)_ = 1.45; *p* = 0.176
		mPFC	*t*_(11)_ = 5.69; *p* < 0.001
	Pretest	Vehicle	*t*_(11)_ = 3.98; *p* = 0.002
		HPC	*t*_(11)_ = 5.41; *p* < 0.001
		mPFC	*t*_(11)_ = 3.20; *p* = 0.008
Figure 3*J* RS79488	Presample	Vehicle	*t*_(11)_ = 5.74; *p* < 0.001
		HPC	*t*_(11)_ = 6.04; *p* < 0.001
		mPFC	*t*_(11)_ = 4.34; *p* = 0.001
	Pretest	Vehicle	*t*_(11)_ = 3.26; *p* = 0.008
		HPC	*t*_(11)_ = 7.13; *p* < 0.001
		mPFC	*t*_(11)_ = 5.03; *p* < 0.001

## Discussion

This study contains several important new findings. We showed, for the first time, that the entire rostrocaudal axis of the NRe is innervated by catecholaminergic fibers and that the LC provides a strong catecholaminergic input to this nucleus. Interestingly the strongest innervation from LC appeared to be to the rostral NRe. Next, optogenetic inactivation of LC→NRe significantly disrupted OiP retrieval, but not encoding, while inactivation of the LC→HPC projection impaired encoding but not retrieval. Finally, we found that retrieval was mediated by increased NA release in the NRe acting at α1-adrenoreceptors, while encoding required NA release in the HPC, specifically acting at β-adrenoceptors. Neither encoding nor retrieval appeared to depend on NA function in the mPFC. While NA release has been associated with attentional processing and arousal (Berridge and Waterhouse, 2003; Berridge, 2008; Sara, 2009; Schwarz and Luo, 2015), significantly none of the optogenetic or pharmacological experimental manipulations disrupted NOR or OL memory. That NOR was not affected is not that surprising given our previous work demonstrating that the HPC, NRe, and mPFC are not involved in this form of recognition memory (Barker and Warburton, 2011, 2018), although the lack of effect on OL does contrast with some earlier findings as will be discussed. Finally, overall object exploration during the sample or test phases was not affected by photostimulation or manipulation of noradrenergic receptor subtypes. Hence, we can exclude the possibility that the observed OiP deficits are due to nonspecific attentional or motivational deficits. Together these findings indicate the importance of NA neuromodulation in discrete brain regions for OiP memory encoding and retrieval.

The significant TH staining across the NRe observed was found to be densest in rostral NRe. These results contrast with an earlier study which found that the catecholaminergic innervation of the midline nuclei, which includes NRe, is sparse (Lindvall et al., 1974). Such differences in findings are likely accounted for by different experimental protocols, as the earlier study used a glyoxylic acid fluorescence method, while here TH was used as the marker. TH is the rate-limiting step of catecholamine biosynthesis and therefore labels both dopaminergic and noradrenergic axons, and while the present study did not distinguish neurochemical identity of these fibers, we revealed that the sole source of potential noradrenergic inputs to the NRe is the LC. However, not all retrogradely labeled cells from the NRe to the LC were TH+; thus, the LC also likely sends noncatecholaminergic inputs to the NRe which may be either GABAergic or glutamatergic (Fung et al., 1994; Nakamura et al., 2000; Glennon et al., 2019; Negishi et al., 2020; Ganley et al., 2021; Yang et al., 2021). Given that our study was in no way meant to be a definitive anatomical investigation of inputs to NRe further studies, including those investigating sources of dopaminergic inputs, are clearly needed. The finding that the rostral NRe has the densest innervation of catecholaminergic fibers is potentially interesting in the context of our previously described OiP memory network, as NRe→HPC projections arise in rostral NRe and projections to mPFC in caudal and lateral wings (Hoover and Vertes, 2012; Varela et al., 2014). Clearly a next step would be to assess whether behavior-specific patterns of neuronal activity occur in HPC-projecting NRe cells modulated by NA.

We next focused on the functional role of LC projections and NA receptor subtypes in the HPC–NRe–mPFC memory network. We consistently found that both disruption of NA signaling in the NRe and disruption of LC input to NRe impaired associative recognition memory retrieval. Changes in behavioral contingencies increase LC firing, thus signaling salience, novelty, or unexpected uncertainty (Vankov et al., 1995; Bouret and Sara, 2005; Yu and Dayan, 2005) as would occur during the OiP test. While the effect of increased LC firing on NRe neurons has not been investigated, in other thalamic nuclei such as the thalamic reticular nucleus, NA increases neuronal excitability through α1-adrenoreceptor activation (McCormick and Prince, 1988; Lee and McCormick, 1996). As associative memory retrieval requires activity in the NRe→HPC pathway (Barker et al., 2021) and the mPFC (Barker et al., 2007), it is tempting to speculate that recognition of a novel object–place arrangement requires top–down mPFC→LC signaling of the object–place change (Schwarz et al., 2015; Breton-Provencher and Sur, 2019) which results in increased LC firing, release of NA in NRe, which acts via α1-adrenoreceptors located specifically on the NRe–HPC projection. Indeed, it has been reported that some LC neurons project to a single brain area and thus have a selective “modular” effect (Kebschull et al., 2016) to optimize behavioral outcomes. NA release in the NRe could thus act to promote ongoing exploration of novelty (Beerling et al., 2011), as one would observe in the OiP task, if retrieval was unaffected.

OiP encoding was disrupted by LC→HPC inhibition, agonism of α2-adrenergic receptors, and antagonism of β-adrenergic receptors. Previous research has shown that novelty, including that during encoding of an object's new location, is associated with LC activation (Kempadoo et al., 2016; Takeuchi et al., 2016; Gálvez-Márquez et al., 2022). Thus, it was surprising that LC→HPC inhibition only impaired OiP and not OL memory which may reflect differences in task difficulty as the OL requires only the single discrimination of the moved object. It has been shown that LC activation releases NA in the HPC, leading to β-adrenergic–dependent synaptic plasticity changes (Hansen and Manahan-Vaughan, 2015; Hagena et al., 2016; Babushkina and Manahan-Vaughan, 2022) specifically long-term depression (Hagena and Manahan-Vaughan, 2025). Such plasticity could provide a mechanism for the longer-term storage of object–place associative memories. However, some recent studies have suggested that projections from the LC to the HPC release dopamine as well as NA, and it is the release of such dopamine rather than NA which is critical for learning and memory (Kempadoo et al., 2016; Takeuchi et al., 2016; McNamara and Dupret, 2017; Wagatsuma et al., 2017). However, using the same protocols, we previously found that direct infusion of the D1/D5 antagonist SCH23390 into the HPC had no effect on OiP encoding (Savalli et al., 2015). Hence overall, our data indicating that NA signaling in the HPC, via β-adrenergic receptors, is required for OiP encoding may reflect the involvement of a NA-mediated underlying long-term synaptic plasticity mechanism ensuring retention of memory over a 3 h delay.

Thus far, encoding and retrieval have been discussed separately, although they are highly dynamic processes and likely to be occurring, on most circumstances, at the same time; thus, we used a modified version of the OiP task involving two test phases (Barker et al., 2021) and confirmed that LC→NRe inactivation impaired retrieval at Test 1, but did not impair encoding of the new information in Test 1, as Test 2 performance was intact. Conversely inactivation of the LC→HPC pathway impaired encoding but not retrieval. These results thus support our proposition that encoding and retrieval are mediated concurrently through separate but parallel LC–forebrain subnetworks, which may be key for the binding of recent and related information while ensuring a separation of processing.

Surprisingly we found no effect of noradrenergic receptor manipulation in the mPFC although the mPFC is pivotal for associative recognition memory (Barker et al., 2007; Barker and Warburton, 2011; Benn et al., 2016), is strongly innervated by noradrenergic fibers, and has dense noradrenergic receptor expression (Palacios and Kuhar, 1982; Scheinin et al., 1994; Rosin et al., 1996; Talley et al., 1996; Paschalis et al., 2009; Santana et al., 2013). Interestingly those functional studies showing the critical role of NA in the mPFC have found effects on short-term working memory and attentional set shifting or in the extinction, but not acquisition, of fear memory (see reviews in Mueller et al., 2008; Berridge and Spencer, 2016), thus underlining the functional and regional specificity of LC and NA signaling in cognition. Indeed recent reports have argued that the LC is a heterogeneous structure where separate populations of LC neurons send selective projections to provide this functional specificity (Chandler and Waterhouse, 2012; Chandler et al., 2014, 2019; Uematsu et al., 2015, 2017; Hirschberg et al., 2017; Giustino et al., 2019; Totah et al., 2019; Borodovitsyna et al., 2020; Ranjbar-Slamloo and Fazlali, 2020). The present data clearly accord with this view, i.e., that during associative recognition memory, LC projections provide localized and hence modular neuromodulation in the NRe and HPC.

These findings demonstrate that memory encoding and retrieval are dependent both on activation of specific pathways and noradrenergic receptor subtypes within a HPC–thalamic memory circuit. Associative recognition memory deficits are associated with several neurodegenerative conditions and neuropsychiatric diseases such as schizophrenia (Mäki-Marttunen et al., 2020; Crawford and Berry, 2024). In aging, the LC cell number and NA concentration in the brain declines (Marien et al., 2004) and in both Parkinson's and Alzheimer's disease LC degeneration occurs relatively early (Braak et al., 2004; Grudzien et al., 2007; Paredes-Rodriguez et al., 2020). Future work should consider a modular LC–NA system in the context of memory circuitry and prevention of memory decline.
